# Effects of High-Intensity Interval and Moderate-Intensity Continuous Exercise on Inflammatory, Leptin, IgA, and Lipid Peroxidation Responses in Obese Males

**DOI:** 10.3389/fphys.2018.00567

**Published:** 2018-05-23

**Authors:** Daniel C. de Souza, Victor A. F. Matos, Victor O. A. dos Santos, Italo F. Medeiros, Cristiane S. R. Marinho, Paulo R. P. Nascimento, Gilson P. Dorneles, Alessandra Peres, Carlos H. Müller, Maurício Krause, Eduardo C. Costa, Ana P. T. Fayh

**Affiliations:** ^1^Post-graduate Program in Physical Education, Health Science Center, Federal University of Rio Grande do Norte, Natal, Brazil; ^2^Department of Nutrition, Health Science Center, Federal University of Rio Grande do Norte, Natal, Brazil; ^3^Health Science College of Trairi, Universidade Federal do Rio Grande do Norte, Natal, Brazil; ^4^Tropical Medicine Institute, Federal University of Rio Grande do Norte, Natal, Brazil; ^5^Laboratory of Cellular and Molecular Immunology, Federal University of Health Sciences of Porto Alegre, Porto Alegre, Brazil; ^6^Research Center, Methodist University Center IPA, Porto Alegre, Brazil; ^7^Laboratório de Pesquisa em Inflamação, Metabolismo e Exercício, Department of Physiology, Institute of Basic Health Sciences, Federal University of Rio Grande do Sul, Porto Alegre, Brazil; ^8^Laboratory of Inflammation, Metabolism, and Exercise Research, Laboratory of Cellular Physiology, Department of Physiology, Institute of Basic Health Sciences, Federal University of Rio Grande do Sul, Porto Alegre, Brazil

**Keywords:** obesity, aerobic exercise, high-intensity interval training, cytokines, immunoglobulin-A, lipoperoxidation

## Abstract

**Purpose:** To compare the effects of a single high-intensity interval exercise session (HIIE) with a moderate-intensity continuous exercise session (MICE) on the inflammatory profile, IgA levels, and lipid peroxidation in sedentary obese males.

**Methods:** Ten sedentary obese men (age 28.5 ± 2.7 years; BMI 35.9 ± 4.9 kg/m^2^; body fat 40.6 ± 2.0%) performed three experimental sessions, on separate days with 1 week wash-out period between interventions, according to a randomized order: (1) HIIE: 10 × 60 s at 90% of the HR_max_ alternated by 60 s of active recovery; (2) MICE: 20 min at 70% of the HR_max;_ (3) Rest—control. Blood and saliva samples were collected before, immediately after and 60 min after the end of each session in order to analyse serum levels of cytokines, IgA, and lipoperoxidation markers.

**Results:** Leptin levels decreased immediately after HIIE (*P* = 0.033) and was different from the MICE (*P* = 0.025). IFN-γ levels were reduced immediately after (*P* = 0.032) and 60 min after HIIE (*P* = 0.003) compared to baseline, and it also increased IL-4 levels immediately after exercise (*P* = 0.007) compared to resting values. MICE promoted an increase in IFN-γ levels immediately after exercise (*P* = 0.025) and 60 min after exercise (*P* = 0.004) in relation to baseline. Both exercise conditions increased IL-6 levels up to 60 min after exercise (*P* < 0.05). The IFN-γ/IL-4 ratio decreased immediately after (*P* = 0.002) and 60 min after HIIE (*P* = 0.005) in relation to pre-exercise. No changes were found for IgA-S and TBARS for any of the conditions.

**Conclusion:** A single HIIE session is able to decrease IFN-γ/IL-4 ratio, indicating an anti-inflammatory response, without alterations in the function of the mucosal immune system and lipoperoxidation. On the other hand, a brief session of MICE induced changes in the pattern of cytokines associated with increased cellular immune function.

## Introduction

Obesity and chronic low-grade inflammation are important risk factors for the development of cardiometabolic diseases (Grundy et al., 2004; Phillips and Prins, 2008). Adipose tissue is considered a complex endocrine organ capable of regulating the immune system and inflammation through the production of adipokines (Galic et al., 2010; Wronska and Kmiec, 2012). Leptin, for example, is an adipokine capable of modulating the activity of proinflammatory phenotype T helper lymphocytes (Th1) (Apostolopoulos et al., 2016) and sympathetic nervous system (da Silva Rossato et al., 2014; Krause et al., 2015). The increase in leptin production seems to trigger an increase in the infiltration of immune cells into visceral adipose tissue (VAT) in response to obesity (Wensveen et al., 2015). Th1 lymphocytes produce interferon-γ (IFN-γ), an important inducer of macrophage polarization to a pro-inflammatory phenotype (M1), and also contribute to the increase of systemic inflammation through the production of inflammatory cytokines such as tumor necrosis factor alpha (TNF-α) (Falcão-Pires et al., 2012; Wensveen et al., 2015).

The production of cytokines by T lymphocytes plays an important role in the adaptive immune response. Th1 (T1) lymphocytes act on the cellular immune response playing a major role in infections, especially against bacteria and viruses. Anti-inflammatory Th2 (T2) phenotype lymphocytes produce interleukin-4 (IL-4) and are responsible for the humoral immune response and prevention of helminth infections and response to allergen agents (Kidd, 2003). The imbalance in the IFN-γ/IL-4 ratio is reported in acute and chronic infections, and it is used as an indicator of the T1/T2 ratio (Zhao et al., 2012). Excess body fat and high levels of pro-inflammatory cytokines are conditions associated with impaired immune systems and greater susceptibility to the development of infectious diseases (Huttunen and Syrjänen, 2012; Kanneganti and Dixit, 2012; Kaspersen et al., 2015, 2016). Moreover, *in vitro* stimulation with leptin in cells of obese individuals resulted in lower IFN-γ production by Natural Killers (NK) cells compared to lean individuals (Laue et al., 2015).

The regular practice of moderate-intensity continuous exercise (MICE) has a positive effect on the inflammatory profile and on the immune system of sedentary obese individuals (Walsh et al., 2011; Trochimiak and Hübner-Wozniak, 2012; Abd El-Kader et al., 2013). Accumulating at least 150 min of MICE per week is recommended for health maintenance in adult subjects (Garber et al., 2011). However, a lack of time is reported as one of the main barriers to the regular practice of physical exercise (Burgess et al., 2017). Thus, high-intensity interval exercise (HIIE) emerges as a time-efficient alternative compared to traditional exercise recommendations as it promotes similar metabolic adaptations to MICE with a lower commitment of weekly time, which may provide greater adherence to the regular practice of physical exercise (Little et al., 2014; Lanzi et al., 2015).

According to previous studies, a single HIIE session is capable of promoting a transient anti-inflammatory state in healthy and overweight individuals, and it can aid in the control of chronic low-grade inflammation (Cabral-Santos et al., 2015; Wadley et al., 2015; Dorneles et al., 2016). Although interleukin-6 (IL-6) is often associated with pro-inflammatory status in individuals with metabolic diseases in resting conditions (Nimmo et al., 2013), its transient increase during and after exercise is recognized as an acute response with metabolic and anti-inflammatory actions, such as inducing an increase in circulating concentrations of anti-inflammatory cytokines IL-4 and IL-10. This action appears to be proportional to the intensity of the effort and the muscle mass involved (Nimmo et al., 2013). In contrast, it is postulated that high-intensity exercise may promote a suppressive effect on the immune response, reducing the secretion rate of salivary immunoglobulin A (IgA-S) and leading to a transient decrease in the IFN-γ/IL-4 ratio, creating an “open window” for infections (Walsh et al., 2011; Zhao et al., 2012). Changes in the immune system are associated with both exercise intensity and duration (Nieman, 1997). As such, a high-intensity lower-volume exercise may promote less impact on the immune function, and it is important to point out that the acute effect of low-HIIE on inflammatory and immunological responses in individuals with obesity is not well-known.

Therefore, the present study aimed to compare the acute effect of a single HIIE session and a time-matched MICE session on the inflammatory profile (leptin, IFN-γ, IL-4, IL-6, and IFN-γ/IL-4 ratio), IgA-S and lipid peroxidation (TBARS) in obese sedentary males.

## Methods

### Study design

This study is characterized as a randomized crossover trial. Sample size was calculated by the statistical power (1—β) with mean and standard deviation of a previous study based on IL-6 (Dorneles et al., 2016), considering a power of 80% and an alpha of 5% (G^*^Power®, version 3.1.9.2; Institute for Experimental Psychology in Dusseldorf, Germany). The study sample consisted of 10 sedentary adult men according to the international physical activity questionnaire (Craig et al., 2003), and obese (body mass index—BMI > 30 m/kg^2^; body fat > 25%) (Pi-Sunyer, 2000; WHO, 2016) non-smokers who reported body weight stability in the last 6 months. The study protocol was approved by the Human Research Ethics Committee at UFRN (Protocol No. 976.389 CAAE 42441015.5.0000.5568) and was registered on the Brazilian site of clinical trials (ReBec registration number: RBR-462kr6f). All subjects gave written informed consent in accordance with the Declaration of Helsinki.

The subjects carried out four visits to the laboratory. During the first visit, the anthropometric measurements, maximal treadmill velocity (MTV), and maximum heart rate (HR_max_) through an incremental treadmill test (RT250, Movement®, Pompeia, Brazil) were collected. Resting blood pressure (BP) was collected according to the Seventh Brazilian Arterial Hypertension Guideline using the oscillometric method (HEM-7200, OMRON, USA). From the second to the fourth visit, the subjects arrived at the laboratory after a night of fasting for approximately 12 h.

**Figure 1:**
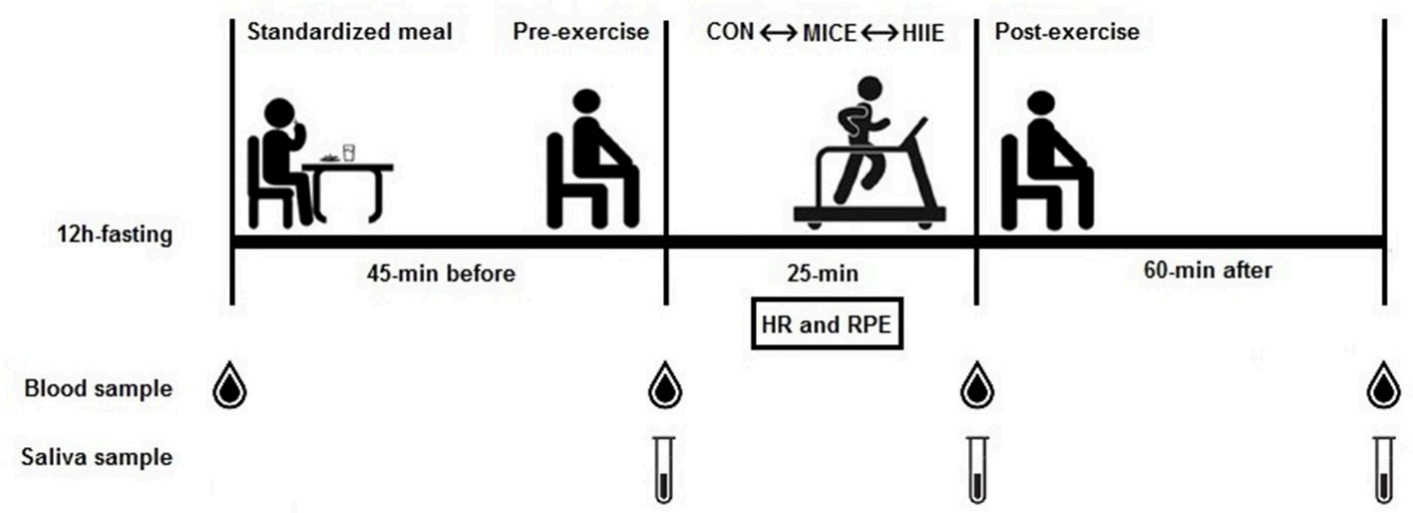
Summary of experimental design. Standardized meal after 12 h fasting; 45-min resting before exercise; 25-min exercise session (control, MICE, and HIIE); 60-min recovery; HR, heart rate; RPE, rating of perceived exertion.

Subjects initially received a standard meal consisting of a nutritional supplement (Hipermassprotein, Atlhética®, Brazil) with an energy content of 4.5 kcal/kg of body weight and diluted in 400 ml of water. Blood collection was performed 45 min after ingesting the standard meal. The subjects were then randomly assigned by a draw to one of three experimental conditions with a 7-day interval (washout) between each session: (1) control; (2) MICE; (3) HIIE (Figure 1).

### Procedures

#### Anthropometric and body composition assessment

The following anthropometric measurements were performed: body mass and height for calculating BMI and nutritional status. Body mass was measured on a digital scale (BC 553, Tanita®, USA) with a maximum capacity of 150 kg and precision of 100 g, with the individual barefoot and wearing light clothes. A portable stadiometer with an accuracy of 1 mm (Personal Caprice Portatil, Sanny®, Brazil) was used to measure height with the individuals in the Frankfurt position. The formula used to calculate BMI was: [BMI = body mass (kg) ^*^ height (m)^−2^]. Double-energy X-ray absorptiometry (DEXA) was also analyzed (GE, Medical Systems, USA), and the participants were instructed to avoid diuretics and caffeinated beverages the day before the evaluation (Albanese et al., 2003).

#### Maximal graded exercise test

The participants performed a warm-up on a treadmill (RT250, Movement®, Brazil) at a speed of 2.0 km/h for 3 min. Then they started the incremental test at a speed of 3.0 km/h and increments of 1.0 km/h every minute until voluntary exhaustion. The MTV was considered as the highest velocity sustained by a full stage of 1 min (Frazão et al., 2016). Heart rate (HR) was monitored throughout the test using a HR monitor (RS800CX, Polar®, Finland) and recorded at the end of each minute. The highest HR value observed during the test was considered as the HRmax. Rating of perceived exertion (RPE) was also monitored and recorded at the end of each minute according to the Borg scale 6–20 (Borg, 1982). The end of the test was determined by the presence of at least one of the following criteria: (i) HR ≥ 100% estimated for age; (ii) RPE > 18; or (iii) when participants voluntarily stopped (Howley et al., 1995).

#### Low-volume HIIE and MICE protocols

HIIE was performed in a 1:1 “effort-recovery.” The participants performed 10 × 1 min work bouts at 90% of their individual MTV reached on the maximal graded exercise test, interspersed by 1 min of active recovery at 30% of MTV (i.e., slow walking). This HIIE model on a treadmill was previously published by our research group (Costa et al., 2016; Frazão et al., 2016; Matos et al., 2017; Fayh et al., 2018). The MICE session consisted of 20 min at 65–75% of HRpeak performed continuously, which is the exercise intensity recommended as moderate by the American College of Sports Medicine physical activity guidelines (Garber et al., 2011). The participants performed a 3 min warm-up at 4 km/h before both exercise sessions, and after the exercise sessions they performed a 2 min cool-down at the same speed. HR was continuously recorded throughout the exercise sessions (RS800CX, Polar®, Finland). In addition, whole-body RPE was assessed using the RPE 6–20 Borg scale (Borg, 1982) during the last 10 s of each minute during both HIIE and MICE. In the control session, the participants remained in a seated position for 25 min and they were allowed to read and use electronic devices such as a tablet, computer, or smartphone.

#### Blood sampling and saliva analyses

Blood samples were obtained from the antecubital vein and from the tip of the finger with the subject sitting in order to determine the lactate for the exercise characterization. At the initial visit, 3 mL of whole blood was collected after a night of approximately 12 h fasting for the sample characterization. Biochemical markers were determined by colorimetric methods using commercial kits (DOLES® kit, Brazil) specific for each parameter. In the experimental sessions, 10 mL of venous blood were collected into tubes with a separator gel for serum (Vacuette®, Greiner Bio-One, Brazil) immediately before the beginning of the session, immediately after the session, and 60 min after the end of the session. During the 60 min post-exercise period, the participants remained in a seated position. Food and drink intake was not allowed. However, they were allowed to read and use electronic devices as in the control condition. The last blood sample was obtained after this period. The blood was centrifuged for 15 min at 3,600 revolutions per minute. The serum was then separated into 200 microliter aliquots and stored at −80°C for further analysis. Concentrations of leptin, IFN-γ, IL-4, and IL-6 were analyzed according to commercial ELISA kits (ELISA Read-Set-Go!®, Ebioscience, USA) following the manufacturer's instructions for analysis on an EZ-Reader microplate reader at 450 nm.

Formation of thiobarbituric acid reactive substances (TBARS) during an acid reaction was used as an index for lipoperoxidation. Samples were first centrifuged at 12,000 g at 4°C for 10 min, then 250 μL of the sample, 10 μL of 4.5 mM butylated Hydroxytoluene (BHT), and 200 μL of 30% trichloroacetic acid (TCA) were added to 1.5 mL eppendorf tubes. These were subsequently placed in a boiling water bath (100°C) for 15 min, and centrifuged at 15,000 g at room temperature for 2 min. Next, 400 μL of supernatant and 400 μL of 0.23% thiobarbituric acid (TBA) were pipetted into the cryotubes and boiled in a 100°C water bath for 30 min. The samples were cooled down for 5 min and pipetted in duplicates of 200 μL into the 96-well plate. TBARS was then determined in a microplate reader at 540 nm. The results were expressed as nmol/mL (Draper and Hadley, 1990).

For the determination of lactate, the tip of the individual's finger was sanitized with a 70% alcohol solution and then pierced with a disposable lancet immediately after the end of the exercise. Approximately 25 μL of blood were collected and analyzed on a portable monitor for lactate determination (Accutrend Plus®, Roche, Switzerland).

Saliva samples were also obtained at the pre-, immediately post-, and 60 min post-exercise with the purpose of determining the salivary IgA-S concentrations at each moment. The saliva was collected without stimulation, with the subjects instructed to rinse their mouths with distilled water and to empty their mouth just before collection. The passive drainage method was used for the collection, in which the volunteer slightly tilted their head forward and allowed the saliva to flow into a pre-weighed and sterilized Falcon tube for a period of 5 min. The tubes were weighed again after collection, so that the volume and the saliva flow rate were estimated. The tubes were weighed with a precision of 0.1 mg with the density of the saliva assumed as (1.0 g.mL^−1^), and the samples were frozen at −80°C for further analysis. The S-IgA levels were analyzed using commercial ELISA kits (IgA Salivary, DRG, USA). The secretion rate of IgA-S (ng/min) represents the total amount of IgA-S present on the mucosal surface per unit of time; for this, the IgA-S concentration (ng/mL) was multiplied by the saliva flow (mL/min).

### Statistical analyses

Data normality was verified through the Shapiro-Wilk test and the homogeneity analysis of the variances through the Levene test. The exercise sessions were compared in relation to the psychophysiological variables through the Student's *t*-test. Two-way analysis of variance (ANOVA) was used (condition vs. time) with repeated measures to compare the inter- and intra-condition effects for dependent variables (Leptin, IFN-γ, IL-4, IL-6, IFN-γ/IL-4 ratio, TBARS, and IgA-S levels). The sphericity hypothesis was verified by the Mauchly test, and the degrees of freedom were corrected by Greenhouse-Geisser estimates when violated. The size of the variance effect was calculated by partial eta-squared (η^2^_*p*_). Additionally, a correlation between the leptin and IFN-γ concentrations was performed for each condition between the different moments through the Pearson correlation. The data were structured and analyzed using the SPSS statistical package (*Statistical Package for Social Sciences Chicago, IL, USA*) version 20.0 for Windows and G^*^ Power version 3.1.9.2 (Institute for Experimental Psychology in Dusseldorf, Germany). All results are expressed as mean and standard deviation, and the accepted level of significance was *P* < 0.05.

## Results

### Subjects and exercise characterization

Subject characteristics are described in Table 1. The results of BMI and fat percentage are compatible with the diagnosis of obesity, while resting BP values indicate that subjects were not hypertensive. Taking into account biochemical analyses at fasting, the subjects, apparently, did not present other significant metabolic alterations.

**Table 1:** Characteristics of the subjects (*n* = 10).

	**Mean ± SD**
Age (years)	28.5 ± 2.7
Weight (kg)	109.3 ± 18.8
Height (m)	1.74 ± 0.08
Body mass index (kg/m^2^)	35.9 ± 4.9
Body fat (%)	40.6 ± 2.0
Subcutaneous fat (%)	40.3 ± 3.7
Visceral fat (%)	51.0 ± 2.2
Peak heart rate (bpm)	197 ± 12
Resting systolic blood pressure (mmHg)	124 ± 8
Resting diastolic blood pressure (mmHg)	79 ± 8
Fasting glucose (mg/dL)	98.2 ± 26.7
Total cholesterol (mg/dL)	202.9 ± 25.3
HDL-cholesterol (mg/dL)	42.8 ± 2.5
LDL-cholesterol (mg/dL)	135.2 ± 27.6
VLDL-cholesterol (mg/dL)	24.8 ± 7.8
Tryglicerides (mg/dL)	124.2 ± 39.2
Aspartate Aminotransferase; (mg/dL)	24.6 ± 6.1
Alanine Aminotrasnferase (mg/dL)	27.4 ± 6.5
Urea (mg/dL)	29.0 ± 8.3
Creatinine (mg/dL)	0.8 ± 0.2
Uric acid (mg/dL)	5.2 ± 1.8
**PHYSICAL ACTIVITY LEVEL**	
Walking (min/week)	17.5 ± 24.4
Moderate activity (min/week)	9.0 ± 15.2
Vigorous activity (min/week)	0 ± 0
Sitting time (h/day)	10.7 ± 2.1

As shown in Table 2, the HIIE session presented higher values of HR and RPE when compared to MICE, taking into account stimulus values (*P* < 0.05). Lactate levels, shortly after the exercise, were higher for HIIE when compared to MICE (*P* < 0.05).

**Table 2:** Heart rate, rating of perceived exertion and blood lactate responses to low-volume high-intensity interval and moderate-intensity continuous exercise.

	**HIIE**	**MICE**	***p*^a^**
**PEAK HEART RATE %**			
Work bouts	89 ± 5	71 ± 5	<0.01
Recovery	79 ± 7	–	
**RATING OF PERCEIVED EXERTION**			
Work bouts	14.7 ± 1.6	11.8 ± 1.1	<0.01
Recovery	12.5 ± 2.2	–	
Post-exercise blood lactate (mmol/L)	13.1 ± 2.6	5.0 ± 1.5	<0.01

*HIIE, high-intensity interval exercise; MICE, moderate-intensity continuous exercise; a, paired t-test*.

### Blood/saliva cytokine responses to HIIE and MICE

**Figure 2:**
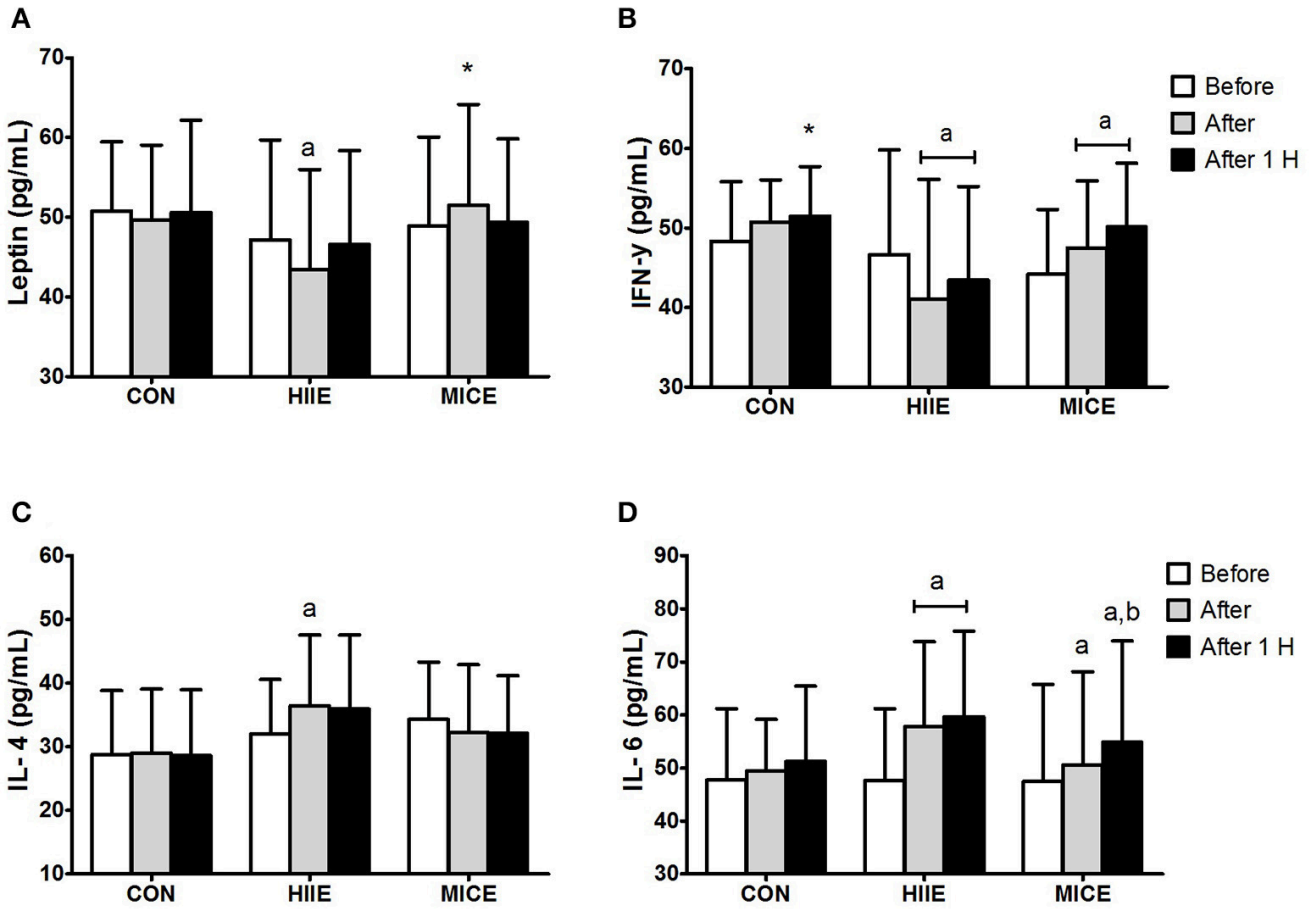
Inflammatory profile before (Before), immediately after (After) and 1 h after (After 1 H) high intensity interval exercise (HIIE), moderate intensity continuous exercise (MICE), and control (CON) condition. Leptin **(A)**; interferon-γ **(B)**; interleukin-4 **(C)**; interleukin-6 **(D)**. Values are presented as mean ± sd. ^a^Significant difference compared with before exercise (*P* < 0.05). ^b^Significant difference compared with before exercise (*P* < 0.05). ^*^Significant difference compared with HIIE (*P* < 0.05).

Figure 2 shows the systemic concentrations of cytokines for the different conditions at the pre-, immediately post-, and 60 min post-exercise. An interaction (condition × time) was observed for leptin [*F*_(4.36)_ = 4.48, *P* = 0.005, $\eta_{p}^{2}$ = 0.33] with a reduction (*P* = 0.033) immediately after HIIE compared to the pre-exercise moment, and a difference between the HIIE and MICE conditions immediately after exercise (*P* = 0.025). An interaction (condition × time) was also observed for IFN-γ [*F*_(4.36)_ = 9.20, *P* = 0.000, $\eta_{p}^{2}$ = 0.506]. IFN-γ decreased (*P* = 0.032) immediately after the HIIE and remained lower than after 60 min compared to pre-exercise (*P* = 0.003). An increase in both post-exercise (*P* = 0.025) and after 60 min (*P* = 0.004) was observed for MICE compared to the pre-exercise moment. Regarding the condition effect, a difference was only found for HIIE after 60 min compared to the control session (*P* = 0.041). An interaction (condition × time) was also observed for IL-4 [*F*_(4.36)_ = 4.30, *P* = 0.006, $\eta_{p}^{2}$ = 0.323] and IL-6 [*F*_(4.36)_ = 3.31, *P* = 0.021, $\eta_{p}^{2}$ = 0.269]. IL-4 levels increased only for HIIE shortly after the session (*P* = 0.007). IL-6 levels increased immediately after (*P* < 0.001) and 60 min after the end of HIIE (*P* < 0.001) and MICE (*P* = 0.031) in relation to pre-exercise. A similar behavior was observed for MICE, both immediately (*P* = 0.031) and 60 min after the exercise (*P* < 0.001). The value of 60 min after MICE also presented a difference in relation to the post-exercise (*P* = 0.033).

**Figure 3:**
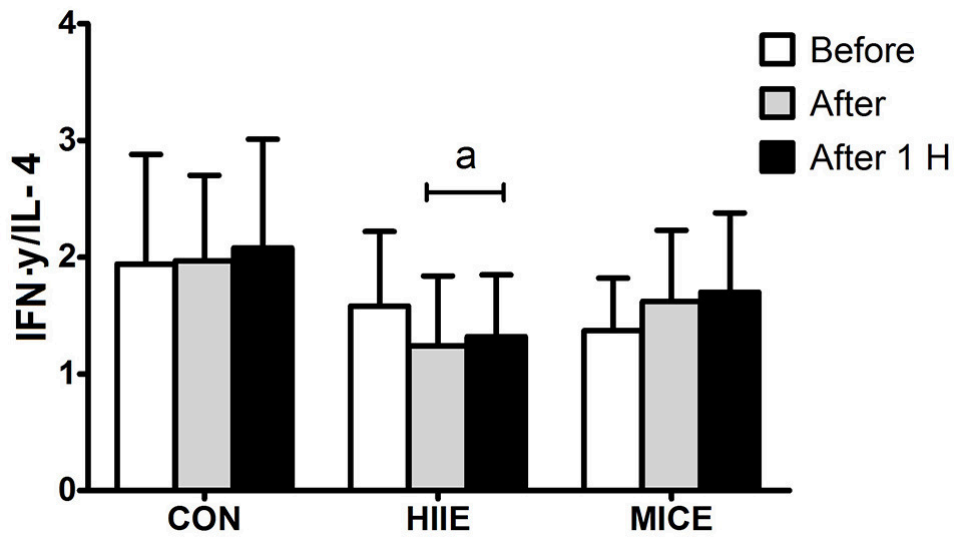
Interferon-γ/interleukin-4 ratio before (Before), immediately after (After) and 1 h after (After 1 H) high intensity interval exercise (HIIE), moderate intensity continuous exercise (MICE), and control (CON) condition. Values are presented as mean ± sd. ^a^Significant difference compared with before exercise (*P* < 0.05).

Figure 3 shows the IFN-γ/IL-4 ratio for the different conditions at before, after and 60 min after exercise moments. An interaction (condition × time) was observed for the IFN-γ/IL-4 ratio [*F*_(4.36)_ = 5.80, *P* = 0.001, $\eta_{p}^{2}$ = 0.392], with a reduction immediately after (*P* = 0.002) and 60 min after (*P* = 0.005) the HIIE in comparison to the pre-exercise. In contrast, the MICE did not promote significant changes in the IFN-γ/IL-4 ratio (*P* > 0.05).

Table 3 shows the IgA-S concentrations and IgA-S secretion rate for the different conditions at before, after and 60 min after exercise moments. No interaction (condition × time) was observed for any of the markers. There was also no interaction for the TBARS values (*P* = 0.518).

**Table 3:** Salivary IgA (S-IgA) concentration, salivary IgA (S-IgA) secretion rate in time before, after, and 1 h after exercise.

	**Before**	**After**	**After 1 H**	***p*^a^**
**S-IgA (ng/mL)**				
CON	138.4 ± 112.1	121.2 ± 20.5	112.4 ± 62.8	0.592
HIIE	136.5 ± 62.4	189.2 ± 186.9	157.5 ± 119.6	
MICE	139 ± 75.3	244.6 ± 192.9	175.7 ± 155	
**S-IgA secretion rate (ng/min)**				
CON	42.4 ± 62.9	45.9 ± 54.3	53 ± 65.1	0.549
HIIE	45.8 ± 65.4	31.3 ± 28.5	54.9 ± 55.9	
MICE	30.3 ± 25.9	51.4 ± 62.4	43 ± 44.1	

CON, control; HIIE, high-intensity interval exercise; MICE, moderate-intensity continuous exercise;

a *Two-way ANOVA with repeated measures*.

In addition, we found positive correlations through the Pearson's correlation between leptin concentrations and IFN-γ concentrations for exercise conditions at before, after and 60 min after exercise moments. No correlation was observed for these two cytokines in the control condition (Table 4).

**Table 4:** Correlation Leptin/IFN-y in different conditions (CON, MICE and HIIE) at moments before, after and 1 h after exercise.

	**Pre**		**Post**		**Post 60 min**	
	***r***	***p***	***r***	***p***	***r***	***p***
CON	0.58	0.077	0.39	0.278	0.43	0.219
MICE	0.77	0.010^*^	0.73	0.015^*^	0.77	0.001^*^
HIIE	0.81	0.004^*^	0.82	0.003^*^	0.72	0.021^*^

* *Statistically significant correlation with Pearson correlation (p < 0.05)*.

## Discussion

Herein, we found that (i) HIIE reduced the leptin levels immediately after the exercise and the IFN-γ/IL-4 ratio up to 60 min post exercise; (ii) MICE increased IFN-γ levels up to 60 min post exercise; (iii) both HIIE and MICE increased the IL-6 levels up to 60 min post exercise; and (iv) no changes in IgA and lipid peroxidation levels were observed following HIIE and MICE. To the best of our knowledge, this is the first study to report changes induced by the HIIE in IFN-γ/IL-4 ratio in sedentary obese males.

IFN-γ is a cytokine mainly produced by NK cells, CD4+ T lymphocytes, and Th1 CD8+, which play a critical role in the innate and adaptive immune response against viruses, some types of bacteria, and protozoa (Schoenborn and Wilson, 2007). Thus, suppression in the production of IFN-γ by T cells after exercise could represent reduced immune defenses against viral and bacterial infections. The reduce in IFN-γ by high-intensity physical exercise may represent a temporary decrease in T1-mediated immunity (Walsh et al., 2011). As previously reported, an incremental interval ergometer test, consisting of 3 min of exercise at maximum power, alternated with 30 s of passive rest, showed a decrease in the *in vitro* soluble levels of INF-γ after stimulation with anti-CD2/anti-CD28 (Smits et al., 1998). Also, the reduced INF-γ levels after strenuous exercise (1.5 h in 5% downhill running at 75% of maximal capacity) was associated with a low peripheral frequency of memory CD8+ T lymphocytes in the peripheral blood, indicating a transient reduction in immunovigilance against intracellular pathogens (Ibfelt et al., 2002).

In the present study, the reduced IFN-γ levels in addition to an increase in IL-4 concentrations immediately after an HIIE session reinforces the hypothesis that T1 and T2 cells exert reciprocal inhibitory actions on each other after an exercise session (Kidd, 2003). The increase of IL-4 may activate B lymphocytes, promoting their proliferation and differentiation into memory and plasma cells. B cells in plasma contribute to the increase in the amount of circulating antibodies, favoring a first line of defense against antigens and pathogens (Kidd, 2003; Walsh et al., 2011). In this sense, a transient increase in IL-4 levels may favor the humoral immune system against the action of extracellular pathogens. Moreover, considering that IL-4 has anti-inflammatory properties, an increase in the concentrations of this cytokine can help in reducing the inflammatory state and stimulate the polarization of macrophages to their M2 anti-inflammatory phenotype (Ignacio et al., 2014; Apostolopoulos et al., 2016).

On the other hand, the reduction in the inflammatory capacity of leukocytes due to exercise may impair immune function. The reduction in the IFN-γ/IL-4 ratio observed in the present study immediately after and maintained up to 60 min after an HIIE session may represent an “open window” for infections associated with decreased cellular immune response in obese individuals (Walsh et al., 2011; Zhao et al., 2012). In contrast, no alterations in IgA levels or in the secretion rate were observed (Table 3). To the best of our knowledge, this is the first study describing the acute influence of different types of exercise on IgA levels in sedentary obese individuals. IgA is an immune mucosal marker and it is considered a first barrier for pathogens. This reduction is related to a higher chance of infections in the upper respiratory tract (Walsh et al., 2011), which could hamper subsequent exercise sessions and thus impair the adherence of individuals who initiate a physical fitness program. Therefore, it is important to point out that our findings suggest that HIIE or MICE session does not compromise the mucosal immunity of inactive and highly sedentary individuals with obesity. In addition, TBARS (an indirect marker of oxidative stress), according to our findings did not increase at the 60 min time point for both exercise types. This is in contrast to the findings of Fisher et al. (2011) who found an increase in TBARS after performing an HIIE protocol. However, this difference may be associated with the type of protocol used in their study (Fisher et al., 2011), as they used HIIE maximal protocol with an “all out” bouts, while the present study used a submaximal HIIE protocol.

It is important to highlight how a low-volume MICE session (i.e., 20 min at ~70% of HRpeak) improved the T1 response pattern up to 60 min after exercise. Considering that obesity is associated with impairments in the cellular immune response against infectious agents (Huttunen and Syrjänen, 2012; Green and Beck, 2017), MICE appears to improve the immune response mediated by T1 cells in obese men. Moreover, MICE also increased the anti-inflammatory response by a transient increase of IL-6 up to 60 min after exercise. Together, these results may suggest that 20 min of MICE seems to induce positive changes on inflammatory profile and immune responses in highly inactive sedentary males with obesity. Chronic low-grade inflammation is an important risk factor for developing diseases of cardiometabolic etiology (Grundy et al., 2004; Phillips and Prins, 2008). In this sense, both an HIIE session and a MICE session seem to modulate the inflammatory state, promoting a transient increase in concentrations of anti-inflammatory cytokines in obese subjects (Højbjerre et al., 2007; Dorneles et al., 2016) as observed in the present study (i.e., elevation of IL-6 levels); however, an additional anti-inflammatory effect was observed after HIIE as a function of suppressing the inflammatory response pattern of lymphocytes due to the reduction in the INF-γ/IL-4 ratio.

Leptin is largely expressed in adipose tissue and is recognized for its role in regulating appetite and metabolic control (Falcão-Pires et al., 2012). In the immunological context, this adipokine is responsible for activating the pro-inflammatory phenotype through increased glucose uptake by Th1 cells mediated by glucose transporter-1 (GLUT-1) activity. In addition, leptin is a known activator of the sympathetic system, that directly causes activation of the immune cells (da Silva Rossato et al., 2014; Krause et al., 2014, 2015). Thus, leptin can be considered the link between adipose tissue, metabolic activity and functioning of effector T cells (Saucillo et al., 2014; Apostolopoulos et al., 2016). Serum leptin levels are associated with the amount of adipose tissue. In obesity, the excess of leptin is recognized for promoting an increase in the amount of immune cells in visceral adipose tissue (Wensveen et al., 2015). As leptin is responsible for activating Th1 lymphocytes (Apostolopoulos et al., 2016), its transient reduction can modulate T cell function during exercise. Interestingly, we observed a correlation between the levels of IFN-γ and leptin levels in exercise conditions for the first time (Table 4), which suggests that adipose tissue, along with sympathetic nervous system, may be able to modulate the cytokine pattern secreted by T cells during exercise.

It is important to emphasize that our study was not able to indicate the expression pattern or the cellular origin of these cytokines, since both T and NK cells synthesize and secrete IFN-γ into the circulation. It should also be pointed out that the inflammatory and immune response can continue 60 min after the end of the exercise. The follow-up in the present study was only performed up to this moment.

Several studies have suggested HIIE as a time-efficient alternative compared to traditional MICE recommendations (Gibala et al., 2012). This type of exercise is able to increase VO_2max_ (Lanzi et al., 2015) and improve risk factors for diseases of metabolic etiology in overweight individuals (Gillen et al., 2013; Little et al., 2014). Thus, this study contributes to narrow the gap in the literature on the effects of a HIIE and MICE on inflammatory markers in obese individuals who do not yet present other metabolic alterations, thus collaborating to optimize the exercise prescription for this population. However, further clarification is needed regarding the clinical relevance of the acute effect of HIIE and MICE on the IFN-γ/IL-4 ratio.

In conclusion, the change in the IFN-γ/IL-4 ratio observed after an HIIE session may indicate an anti-inflammatory response, however without compromising the mucosal immune system or increasing oxidative stress. On the other hand, a brief MICE session induced changes in cytokine pattern associated with improved cellular immune response in obese males.

## Author contributions

DdS, VM, VdS, CSRM, GD, AP, CM, MK, EC, and AF contributed conception and design of the study. DdS, VM, VdS, PN, and CM organized the database. DdS, VM, VdS, IM, CSRM, CM, and EC performed the statistical analysis. DdS, GD, AP, MK, EC, and AF wrote the first draft of the manuscript. DdS, CSRM, GD, AP, MK, EC, and AF wrote sections of the manuscript. All authors contributed to manuscript revision, and they read and approved the submitted version.

### Conflict of interest statement

The authors declare that the research was conducted in the absence of any commercial or financial relationships that could be construed as a potential conflict of interest.
